# Characterization of Sleep Breathing Pattern in Patients with Type 2 Diabetes: Sweet Sleep Study

**DOI:** 10.1371/journal.pone.0119073

**Published:** 2015-03-11

**Authors:** Albert Lecube, Gabriel Sampol, Cristina Hernández, Odile Romero, Andreea Ciudin, Rafael Simó

**Affiliations:** 1 CIBER de Diabetes y Enfermedades Metabólicas Asociadas (CIBERDEM), Instituto de Salud Carlos III (ISCIII), Endocrinology Department, Diabetes and Metabolism Research Unit, Institut de Recerca i Hospital Universitari Vall d’Hebron (VHIR), Universitat Autònoma de Barcelona, Barcelona, Spain; 2 Endocrinology and Nutrition Department, Hospital Universitari Arnau de Vilanova, IRB-Lleida, Universitat de Lleida, Lleida, Spain; 3 CIBER Enfermedades Respiratorias (CIBERES), Instituto de Salud Carlos III (ISCIII), Sleep Unit, Neurophysiology Department, Institut de Recerca i Hospital Universitari Vall d’Hebron (VHIR), Universitat Autònoma de Barcelona, Barcelona, Spain; Weill Cornell Medical College in Qatar, QATAR

## Abstract

**Background:**

Although sleep apnea-hypopnea syndrome (SAHS) is highly prevalent in patients with type 2 diabetes (T2D), it is unknown whether or not subjects with and without T2D share the same sleep breathing pattern.

**Methodology/Principal findings:**

A cross-sectional study in patients with SAHS according to the presence (n = 132) or not (n = 264) of T2D. Both groups were matched by age, gender, BMI, and waist and neck circumferences. A subgroup of 125 subjects was also matched by AHI. The exclusion criteria included chronic respiratory disease, alcohol abuse, use of sedatives, and heart failure. A higher apnea hypopnea index (AHI) was observed in T2D patients [32.2 (10.2–114.0) *vs*. 25.6 (10.2–123.4) events/hours; p = 0.002). When sleep events were evaluated separately, patients with T2D showed a significant increase in apnea events [8.4 (0.1–87.7) *vs*. 6.3 (0.0–105.6) e/h; p = 0.044), as well as a two-fold increase in the percentage of time spent with oxygen saturation <90% [15.7 (0.0–97.0) *vs*. 7.9 (0.0–95.6) %; <0.001)], higher rates of oxygen desaturation events, and also higher daily sleepiness [7.0 (0.0–21.0) *vs*. 5.0 (0.0–21.0); p = 0.006)] than subjects without T2D. Significant positive correlations between fasting plasma glucose and AHI, the apnea events, and CT90 were observed. Finally, multiple linear regression analyses showed that T2D was independently associated with AHI (R^2^ = 0.217), the apnea index (R^2^ = 0.194), CT90 (R^2^ = 0.222), and desaturation events.

**Conclusions/significance:**

T2D patients present a different pattern of sleep breathing than subject without diabetes. The most important differences are the severity of hypoxemia and the number of apneas whereas the incidence of hypopnea episodes is similar.

## INTRODUCTION

Sleep apnea-hypopnea syndrome (SAHS) is well established as an independent risk factor for hypertension, myocardial infarction, and stroke [1]. In addition, the available data suggest that a long-term exposure to intermittent hypoxia and sleep fragmentation also contribute to disorders of glucose metabolism [2]. In this regard, a high prevalence of fasting hyperglycemia, insulin resistance, and type 2 diabetes mellitus (T2D) has been found among SAHS patients in comparison with healthy subjects [3, 4].

Alternatively, because of its considerable vascularization and abundant collagen and elastin fibers, the lung parenchyma suffers the harmful effects of T2D, and both insulin resistance and chronic hyperglycemia have been proposed as contributors to the development of SAHS [5]. An analysis of the *Sleep Heart Health Study* found that patients with T2D had increased sleep-disordered breathing and more severe sleep hypoxemia, although after correcting for the main factors involved in the development of SAHS this difference in sleep hypoxemia was eliminated [6]. Additionally, in non-obese rats, Ramadan *et al*. [7] demonstrated that insulin resistance was one of the primary mechanisms leading to sleep apnea, and that treatment with metformin, a drug currently used to increase insulin sensitivity, reversed and prevented these episodes. Recently, we have contributed to demonstrating that T2D is an independent risk factor for severe nocturnal hypoxemia and the impairment of lung function parameters [8, 9]. These data are significant because the impairment of respiratory capacity and nocturnal hypoxia are well-recognized cardiovascular risk factors which can be added to the classic cardiovascular risk factors associated with T2DM [10, 11].

However, it is unknown whether or not patients with T2D share the same sleep breathing pattern as subjects without diabetes. On this basis, the aim of this cross-sectional study was to compare the characteristics of polysomnography records in patients with SAHS according to the presence of T2D. Both groups were closely matched by the most important variables that could affect the prevalence and severity of sleep-disordered breathing.

## MATERIALS AND METHODS

### Ethic statement

Informed written consent was obtained from all participants, and the human ethics committee from the Hospital Universitari Vall d’Hebrón approved the study.

### Description of patients

This was a retrospective study investigating the effect of T2D on the sleep breathing pattern. A total of 732 patients with T2D evaluated for suspected SAHS at the Sleep Unit of our university hospital between January 2012 and August 2013 were screened. Inclusion criteria included: age older than 18 years, Caucasian origin, known type 2 diabetes for longer than 5 years, non-active tobacco smoking, an apnea hypopnea index (AHI) higher than 10 events/hour, and polysomnographic studies with more than 5 hours of correct signal recording.

Among the 244 patients who met these criteria, 113 were excluded. Reasons for exclusion were the following: chronic pulmonary disease (n = 39), narcolepsy (n = 4), stroke (n = 15), malignancy (n = 11), heart failure (n = 16), abuse of alcohol (> 40 g/day for men and > 20 g/day for women) or use of sedatives (n = 8), goitre with obstructive or compressive symptoms (n = 6), hemoglobin lower that 11g/dL (n = 3), treatment with steroids (n = 14), chronic renal failure (n = 6), and clinical manifestations of diabetic autonomic neuropathy (ie. orthostatic hypotension, gastroparesis diabeticorum, fecal incontinence, neurogenic bladder, anhidrosis or severe erectile dysfunction) (n = 1). No pregnant women were evaluated. A complete physical examination was performed (with special attention to neurological, cardiopulmonary, and ear, nose and throat evaluations), and a chest radiography was performed on all patients included in the study.

We aimed to select two controls for every patient with T2D and, consequently, 262 subjects without diabetes evaluated at the same Sleep Unit served as a control group. Both groups were closely matched by age, gender, body mass index (BMI), and waist and neck circumferences. Similarly to patients with diabetes, only polysomnographic studies with more than 5 hours of correct signal recording were included in the control group.

Treatment for T2D was heterogeneous, and included combined treatment with metformin plus basal insulin (17.5%), basal plus or basal bolus therapy (13.7%), metformin plus sulphonylureas (29.0%), metformine alone (11.4%), and diet (4.5%). The remaining thirty-one patients (23.6%) were under different oral treatment combinations including thiazolidinediones, dipeptidyl peptidase-4 inhibitors, and human glucagon like peptide-1 analogues. The main clinical characteristics and metabolic data of the study population are shown in Table 1. However, previous groups were not equally by the AHI, a known potential confounding factor when metrics of SAHS are evaluated. Therefore, 125 from the 132 patients with T2D were also matched (1:1) to 125 controls not only by the previous conditions but also by the AHI.

**Table 1:** Main clinical characteristics and metabolic data of participants included in the study according to the presence of type 2 diabetes.

	T2D	Non T2D	Mean difference (95% CI)	p value
**n**	131	262	-	-
**Age (yrs)**	53.3 ± 11.2	52.3 ± 10.6	1.2 (−1.0 to 3.5)	0.279
**Women (%)**	62.6	62.6	-	-
**BMI (Kg/m** ^**2**^)	41.3 ± 8.8	42.0 ± 8.5	−0.7 (−2.5 to 1.1)	0.450
**Neck circumference (cm)**	41.7 ± 3.0	41.2 ± 3.6	0.4 (−0.7 to 1.6)	0.445
**Waist circumference (cm)**	123.5 ± 16.9	120.9 ± 16.7	2.6 (−1.9 to 7.1)	0.258
**Fasting glucose (mg/dl)**	167.0 ± 53.4	98.6 ± 11.1	68.3 (3.6 to 61.2)	<0.001
**HbA1c (%)**	7.8 ± 1.6	5.7 ± 0.3	2.1 (0.1 to 1.8)	<0.001
**HbA1c (mmol/mol)**	62.0 ± 17.5	39.0 ± 3.3	23.0 (1.1 to 19.7)	<0.001

BMI: body mass index; HbA1c: glycated hemoglobin

### Measurement of sleep disorders of breathing

Overnight sleep study was conducted in a private room at the Sleep Unit using a computerized polysomnography device (Compumedics E Series, Abbotsdorf, Australia). Polysomnography included recordings of six electroencephalographic channels, bilateral electro-oculograms, chin and tibial electromyogram, electrocardiogram, airflow by nasal pressure transducer and oronasal thermocouples, as well as oxygen saturation by finger pulse oximeter. An expert scorer blinded to the study reviewed all sleep studies manually. An apnea was defined as the cessation of airflow with duration of at least 10 seconds. Differentiation was made between obstructive and central apneas according to the respiratory effort channels (presence or absence of thoracoabdominal movements). Hypopnea was defined as a >30% reduction in nasal cannula tracing with a duration of at least 10 seconds associated with a cyclical dip in arterial oxygen saturation (SaO_2_) ≥ 3%. The AHI was defined as the sum of apneas plus hypopneas divided by the total hours of sleep. On this basis, SAHS was defined as an AHI ≥ 10 events/hour, and patients were divided into non-SAHS (AHI < 10 events/hour), mild SAHS (AHI between 10 and 20 events/hour), moderate SAHS (AHI between 21 and 29 events/hour), and severe SAHS (AHI >30 events/hour). Similarly, the apnea index (AI) and the hypopnea index (HI) were defined as the number of apnea or hypopnea events divided by the total hours of sleep. Three oxygen saturation measures were assessed: the cumulative percentage of time spent with oxygen saturations below 90% (CT90), and the basal and the average SaO_2_. In addition, the oxygen desaturation index (ODI) per hour was calculated by dividing the total number of oxygen desaturation events by the total hours of sleep. Four different oxygen desaturation thresholds (reductions in SaO_2_ equal or greater than 2%, 3%, 4%, and 5%) as indicators of hypoxemia severity (ODI-2%, ODI-3%, ODI-4%, and ODI-5%) were also determined.

The degree of sleepiness was assessed using the Epworth Sleepiness Scale (ESS), a widely used questionnaire based on the tendency to fall asleep during various daytime situations [12]. A score of 10 or more is considered sleepy.

T2D was defined according to the criteria recommended by the Expert Committee on the Diagnosis and Classification of Diabetes [13]. Sixty-three of the 131 patients with T2D were under metformin treatment alone, whereas fifteen patients were under diet counseling alone. The remaining subjects were under mixed therapies including other oral and subcutaneous antidiabetic agents.

Fasting plasma glucose [hexoquinase method (Olympus Diagnostica GmbH, Hamburg, Germany)] was obtained from all subjects with and without T2D. HbA1c (chromatography) was obtained from all patients with T2D and 82.0% (n = 215) of subjects without diabetes.

### Statistical analysis

The normal distribution of the variables was evaluated using the Kolmogorov-Smirnov test. Data were expressed either as the mean ± SD, percentage or median (total range). For parametric tests, AHI, AI, HI, and CT90 were logarithmically transformed to achieve a normal distribution. Comparisons between groups were performed using the Student’s *t* test or the Mann-Whitney U test for continuous variables, as well as the χ^2^ test for categorical variables.

The relationship between the continuous variables was examined by the Pearson linear correlation test. Several stepwise multiple linear regression analyses to explore the variables independently related to measurements of sleep breathing (AHI, AI, HI, CT90, average and minimum SaO_2_, ODI-2%, ODI-3%, ODI-4%, and ODI-5%) were performed in the whole population. The independent variables included in the analyses were: gender, age, BMI, basal SaO_2_, neck circumference, waist circumference, somnolence score, and the presence or absence of T2D. All “p” values were based on a two-sided test of statistical significance. Significance was accepted at the level of p<0.05. Statistical analyses were performed using the SSPS statistical package (SPSS, Chicago, IL, USA).

## RESULTS

The polysomnographic parameters of the study population are presented in Table 2. Patients with T2D showed a higher AHI than subjects without diabetes [32.2 (10.2–114.0) *vs*. 25.6 (10.2–123.4) events/hour; p = 0.002]. In addition, a higher prevalence of severe SAHS was observed in patients with T2D (54.2% vs. 41.9%; p = 0.008). When sleep events were evaluated separately, a similar frequency of hypopnea events was present in patients with and without diabetes [16.5 (0.0–75.3) *vs*. 15.1 (0.0–71.4) e/h; p = 0.285]. However, patients with T2D showed a higher number of apnea events [8.4 (0.1–87.7) *vs*. 6.3 (0.0–105.6) e/h; p = 0.044]. No differences in the number of either central or obstructive apnea events were observed in our study.

**Table 2:** Pulmonary parameters of participants included in the study according to the presence of type 2 diabetes.

	T2D	Non T2D	p value
**n**	131	262	-
**AHI (events/hour)**	32.2 (10.2–114)	25.6 (10.2–123.4)	0.002
**Apnea index (events/hour)**	8.4 (0.1–87.7)	6.3 (0.0–105.6)	0.044
**Hypopnea index (events/hour)**	16.5 (0.0–75.3)	15.1 (0.0–71.4)	0.285
**Basal SaO**_**2**_ **(%)**	96.0 (86.0–100.0)	95.0 (56.0–100.0)	0.646
**Average SaO**_**2**_ **(%)**	92.0 (75.0–98.0)	93.0 (81.0–100.0)	0.026
**Minimum SaO**_**2**_ **(%)**	71.0 (37–98)	75.0 (37.5–97.0)	0.033
**CT90 (%)**	15.7 (0.0–97.0)	7.9 (0.0–95.6)	0.006
**ODI 2% (events/hour)**	42.2 (8.0–284.0)	33.0 (0.0–129.1)	0.014
**ODI 3% (events/hour)**	35.4 (7.0–278)	26.0 (0.0–123.0)	0.037
**ODI 4% (events/hour)**	28.6 (1.9–221.0)	18.4 (0.0–121.0)	0.014
**ODI 5% (events/hour)**	21.9 (0.8–186.0)	12.5 (0.0–115.0)	0.010
**EES**	7.0 (0–21.0)	5.0 (0–21.0)	<0.001

AHI: apnea-hypopnea index; SaO2: oxygen arterial saturation; CT90: cumulative percentage of time spent with oxygen saturation below 90%; ODI: oxygen desaturation index; EES: Epworth Sleepiness Scale score.

Patients with T2D spent a two-fold higher percentage of time with oxygen saturation below 90% in comparison with subjects without diabetes [15.7 (0.0–97.0) *vs*. 7.9 (0.0–95.6) %; p<0.001). Similarly, the average SaO_2_ was lower in patients with T2D [92.0 (75.0–98.0) vs. 93.0 (81.0–100.0) %; p = 0.026], as well as the minimum SaO_2_ [71.0 (37.0–98.0) *vs*. 75.0 (37.5–97.0)%; p = 0.033], and higher rates of ODI events were observed in patients with T2D. Regarding somnolence, patients with T2D showed higher daily sleepiness [7.0 (0.0–21.0) vs. 5.0 (0.0–21.0); p = 0.006] than subjects without diabetes.

When the prevalence of apnea and hypopnea events was separately evaluated in the subgroup of patients also matched by the AHI, patients with T2D still presented a significantly higher number of apnea episodes [7.4 (0.1–85.7) vs. 5.0 (0.0–105.6); p = 0.036] in comparison subjects without diabetes (Table 3). However, between both subgroups, no differences in oxygen measurements were observed (Table 4).

**Table 3:** Main clinical characteristics, metabolic data, and pulmonary parameters when patients with T2D and subjects without diabetes are matched not only by age, gender, BMI, and neck and waist circumferences, but also by AHI.

	T2D (n = 125)	Non T2D (n = 125)	Mean difference (95% CI)	p value
**Age (yrs)**	53.7±11.1	51.5±9.2	2.2 (−0.3 to 4.7)	0.091
**Women (%)**	60.8	60.8	-	-
**BMI (Kg/m** ^**2**^)	40.8±8.6	42.0±7.4	−1.1 (−3.1 to 0.8)	0.266
**Neck circumference (cm)**	41.5±2.8	41.8±3.5	−0.3 (−1.6 to 0.9)	0.622
**Waist circumference (cm)**	122.6±16.9	122.8±14.2	−0.1 (−5.0 to 4.7)	0.952
**Fasting glucose (mg/dl)**	166.2±52.4	98.5±11.0	67.6 (57.6 to 77.6)	<0.001
**HbA1c (%)**	7.8±1.6	5.7±0.3	2.1 (1.7 to 2.4)	<0.001
**AHI (events/hour)**	31.3 (10.2–106.3)	31.1 (10.3–106.3)	-	0.934
**Apnea index (events/hour)**	7.4 (0.1–85.7)	5.0 (0.0–105.6)	-	0.036
**Hypopnea index (events/hour)**	16.1 (0.0–75.3)	20.2 (0–71.4)	-	0.005
**Basal SaO**_**2**_ **(%)**	96.0 (86.0–100.0)	95.0 (56.0–100.0)	-	0.154
**Average SaO**_**2**_ **(%)**	93.0 (75.0–98.0)	93.0 (81.0–97.0)	-	0.820
**Minimum SaO**_**2**_ **(%)**	72.0 (37.0–98.0)	75.0 (37.0–97.0)	-	0.070
**CT90 (%)**	14.2 (0.1–97.0)	10.9 (0.1–95.0)	-	0.188
**ODI 2% (events/hour)**	41.5 (8.0–172.6)	42.0 (9.9–105.6)	-	0.949
**ODI 3% (events/hour)**	31.6 (7.0–278.0)	33.5 (0.0–98.0)	-	0.913
**ODI 4% (events/hour)**	25.5 (1.9–206.3)	24.8 (0.0–98.0)	-	0.828
**ODI 5% (events/hour)**	18.8 (0.8–106.0)	17.2 (0.0–97.0)	-	0.659

AHI: apnea-hypopnea index; BMI: body mass index; HbA1c: glycated hemoglobin

**Table 4:** Stepwise multiple linear regression analysis of variables associated with parameters obtained from the polysomnographic study in the whole population (n = 393 subjects).

		Beta	Beta 95% IC	p value
**AHI (log)**	Neck circumference (cm)	0.379	0.016 to 0.042	<0.001
	Type 2 diabetes (yes/no)	−0.223	−0.247 to −0.030	0.013
R^2^ = 0.217	*Constant*	-	−0.108 to 1.119	0.105
**Apnea index (log)**	Gender (men/women)	0.357	0.276 to 0.823	<0.001
	Type 2 diabetes (yes/no)	−0.240	−0.708 to −0.106	0.009
R^2^ = 0.194	*Constant*	-	0.694 to 1.826	<0.001
**Hypopnea index (log)**	BMI (kg/m^2^)	0.271	0.003 to 0.021	0.007
R^2^ = 0.168	*Constant*	-	0.309 to 0.980	<0.001
**CT90 (log)**	Neck circumference (cm)	0.244	0.013 to 0.083	0.008
	Type 2 diabetes (yes/no)	−0.278	−0.718 to −0.160	0.002
	Basal SaO_2_ (%)	−0.227	−21.215 to −2.618	0.013
R^2^ = 0.221	*Constant*	-	4.496 to 42.020	0.016
**Average SaO**_**2**_ **(log)**	Basal SaO_2_ (%)	0.391	0.226 to 0.592	<0.001
	Neck circumference (cm)	−0.225	−0.002 to 0.000	0.012
R^2^ = 0.234	*Constant*	-	0.826 to 1.562	<0.001
**ODI-2% (log)**	Type 2 diabetes (yes/no)	−0.280	−0.327 to −0.060	0.005
	Neck circumference (cm)	0.278	0.007 to 0.040	0.005
R^2^ = 0.178	*Constant*	-	0.044 to 1.527	0.038
**ODI-3% (log)**	Neck circumference (cm)	0.314	0.013 to 0.048	0.001
	Type 2 diabetes (yes/no)	−0.235	−0.336 to −0.047	0.012
R^2^ = 0.175	*Constant*	-	−0.347 to 1.264	0.261
**ODI-4% (log)**	Neck circumference (cm)	0.290	0.012 to 0.054	0.002
	Type 2 diabetes (yes/no)	−0.249	−0.400 to −0.062	0.008
R^2^ = 0.164	*Constant*	-	−0.618 to 1.267	0.496
**ODI-5% (log)**	Neck circumference (cm)	0.306	0.015 to 0.062	0.001
	Type 2 diabetes (yes/no)	−0.236	−0.446 to −0.066	0.009
R^2^ = 0.169	*Constant*	-	−1.033 to 1.091	0.957

The independent variables included in all analyses were: gender, age, BMI, basal SaO_2_ (log), neck circumference, waist circumference, somnolence score, and the presence or absence of T2D. AHI: apnea-hypopnea index; Beta: Standardized partial regression coefficient. ODI: oxygen desaturation index; BMI: body mass index.

Univariate analysis showed that fasting plasma glucose was positively correlated with AHI (log) (r = 0.147, p = 0.005), the apnea index (log) (r = 0.111, p = 0.039), and CT90 (log) (r = 0.150, p = 0.006). However, no significant correlation was observed between those parameters and HbA1c.

Finally, multiple linear regression analyses showed that the presence of T2D was independently associated with AHI (beta = −0.003, p = 0.013, R^2^ = 0.217), the apnea index (beta = −0.240, p = 0.009, R^2^ = 0.194), CT90 (beta = −0.279, p = 0.002, R^2^ = 0.222), ODI 2% (beta = −0.280, p = 0.005, R^2^ = 0.178), ODI 3% (beta = −0.239, p = 0.010, R^2^ = 0.175), ODI 4% (beta = −0.249, p = 0.008, R^2^ = 0.164), and ODI 5% (beta = −0.246, p = 0.009, R^2^ = 0.169) (Table 4).

## DISCUSSION

In the present study we provide evidence that T2D presents a characteristic pattern of breathing during sleep, with increased apnea events but with no differences in hypopnea episodes. In addition, our results showing an impairment of nocturnal oxygen saturation and daily sleepiness in T2D, reinforce and extend previous reports [9, 14]. Overall, our results suggest that patients with T2D have a more severe sleep breathing disorders than subjects without diabetes. This is is an important finding because sleep related hypoxia is a major stimulus leading to oxidative stress and endothelial dysfunction [15, 16], and may contribute to the increased risk of fatal and non-fatal cardiovascular events observed in T2D. Finally, at the same level of AHI, patients with T2D still present a higher number of apnea events, with a consequent decrease in hypopnea events, but without changes in oxygen saturation measures.

The mechanisms by which T2D patients with SAHS present more severe episodes of airflow reduction during sleep breathing continue to be not fully understood. It has been suggested that there is an interaction between pulmonary function, adipose tissue, and systemic inflammation. Therefore, the increased levels of cytokines (i.e., IL-6 and TNF-alpha) that activate nuclear factor kappa B and the releasing of free fatty acids by excess adipose tissue might be involved in the pathogenic mechanisms leading to apnea in humans [17, 18]. Data supporting this hypothesis include how circulating levels of soluble TNF-alpha soluble receptor (sTNF-R) type 1, but not sTNF-R type 2, were related to reduced lung volumes and airflow limitation in morbidly obese patients prior to the development of a clinically recognized respiratory disease [18]. Similarly, the administration of etanercept, a TNF-alpha antagonist, achieved a significant reduction of sleepiness and sleep apnea and hypopnea events in obese patients with severe SAHS [19].

Metabolic pathways related to insulin resistance have also been advocated as being crucial in initiating lung abnormalities in subjects without diabetes [20]. In this regard, Ramadan *et al* [17] demonstrated that insulin resistance was one of the primary mechanisms leading to sleep apnea in rats, and that treatment with metformin, an insulin-sensitizing drug, not only prevented but also reversed apneic episodes.

The deleterious effect of diabetes on the central respiratory system has also been described [21]. An impairment of ventilatory drive associated with leptin resistance has been shown in obese patients [22]. These clinical observations are supported by experimental studies performed in streptozotocin-induced diabetic rats showing a significant reduction in the ventilatory response to hypercapnic and hypoxic challenges which was prevented with insulin treatment [23]. Resnick *et al* [6], using cross-sectional data from the *Sleep Heart Health Study*, after adjustment for BMI and other potential confounders, found that patients with diabetes had more episodes of periodic breathing, an abnormality of the central control of ventilation, than did those subjects without diabetes. No differences in the number of central episodes between subjects with and without T2D were observed in our study. However, our results raise the central issue of whether the normalization of blood glucose levels in humans can significantly improve the severity of sleep apnea.

Finally, autonomic neuropathy has been implicated in the relationship between T2D and sleep apnea. In fact, T2D patients with dysautonomia were more likely to have not only central, but also obstructive sleep apnea, than those without it [21]. Therefore, an impairment of the upper airway reflexes, possibly due to alterations to the autonomic nervous fibers involved in their regulation, could lead to an inability of individuals with diabetes to respond appropriately to nocturnal airflow reduction episodes, leading to more severe intermittent hypoxic episodes. However, clinical disautonomy was within the exclusion criteria in the present study.

There are some potential limitations that should be taken into account in evaluating the results of our study. One limiting factor is that we recruited a population of mainly obese patients with T2D, and consequently, further studies including a wider spectrum with overweight and lesser degrees of obesity are needed. Second, this was a cross-sectional study and, therefore, a causal link between T2D and the severity of SAHS cannot be drawn. In this regard, research addressed to determining whether the normalization of blood glucose levels could improve SAHS events or hypoxia is warranted. Third, diabetes treatment may influence different conditions that may alter the sleep pattern, and therefore polysomnographic measurements. However, as subjects with T2D were not recruited on the basis of their treatment, almost all of them were under combined management with subcutaneous and oral agents. Consequenly, data about the relationship between baseline therapy and sleep breathing measures are difficult to establish, indicating that further studies to address this possibility are also needed. Finally, although we were able to establish two large and well-balanced groups matched on the basis of the known risk factors that could affect the prevalence and severity of sleep-disordered breathing, there may still be residual confounding factors that may contribute to our findings, such as the prevalence of depression or data regarding the evolution of T2D. Therefore, it is possible that these residual or unmeasured confounders affect some of the results observed in our study.

In conclusion, T2D patients present a different pattern of sleep breathing which is characterized by higher rates of sleep apneas, thus leading to more severe nocturnal hypoxemia and daily sleepiness. However, the number of episodes of hypopnea is very similar to that observed in subjects without diabetes. Whether patients with T2D need to be considered as a vulnerable group, with special attention being paid screening for SAHS, and whether glycemic optimization could be useful in improving the sleep breathing disorders are questions which remain to be elucidated.
